# Evaluation of the Short- and Long-Term Effectiveness of Pulsed Radiofrequency and Conventional Radiofrequency Performed for Medial Branch Block in Patients with Lumbar Facet Joint Pain

**DOI:** 10.1155/2018/7492753

**Published:** 2018-11-22

**Authors:** Abdurrahman Çetin, Abdulkadir Yektaş

**Affiliations:** ^1^Department of Neurosurgery, Republic of Turkey Health Science University, Diyarbakır Gazi Yaşargil Training and Research Hospital, Diyarbakır, Turkey; ^2^Department of Anesthesiology and Reanimation, Republic of Turkey Health Science University, Diyarbakır Gazi Yaşargil Training and Research Hospital, Diyarbakır, Turkey

## Abstract

**Background:**

Diagnosis of lumbar facet joint disease is the sum of the combinations consisting of history, physical activity, and diagnostic imaging frequently including computed tomography and magnetic resonance imaging scans. Prevalence of facet-based chronic low back pain is 15–45%. Intra-articular injections with corticosteroid or medial branch block are traditionally used prevalently in the management of chronic low back pain due to lumbar facet joints. However, the evidence levels of these procedures are at either a low or a medium level. Radiofrequency neurolysis of the lumbar medial branch can be used as an alternative in the management of lumbar facet joint pain. There are two types of radiofrequency applications for radiofrequency neurolysis as pulsed radiofrequency and conventional radiofrequency.

**Materials and Methods:**

Patients with lumbar facet pain were separated into 2 groups. Group 1 (*n*=75): patients were given pulsed radiofrequency under fluoroscopy. Group 2 (*n*=43): patients were given conventional radiofrequency under fluoroscopy. Pre-op and post-op 1^st^, 3^rd^, and 6^th^ month and 1^st^ and 2^nd^ year Visual Analogue Scale values of all patients were asked, recorded, and statistically compared. Visual Analogue Scale values of the groups in the same months were compared as well. At the end of the second year, Odom criteria of both groups were recorded and statistically compared.

**Results:**

Preoperation Visual Analogue Scale values and postoperation 1^st^, 3^rd^, and 6^th^ month and 1^st^ and 2^nd^ year Visual Analogue Scale values were compared in Group 1 and Group 2, and there was a statistically significant difference between preoperation Visual Analogue Scale values and postoperation 1^st^, 3^rd^, and 6^th^ month and 1^st^ and 2^nd^ year Visual Analogue Scale values in both groups. However, the number of repetitions of the operation was higher in Group 1. In the comparison of Odom criteria for both groups at the end of the second year, it was observed that the patients in Group 2 were more satisfied with the treatment.

**Conclusion:**

Conventional radiofrequency in patients with lumbar facet joint pain for medial branch neurolysis effectively decreases Visual Analogue Scale values in both short and long term. The quality of life and daily activities of patients were better at conventional radiofrequency.

## 1. Introduction

Chronic low back pain (CLBP) is a reason for disability [1]. Lumbar intervertebral discs, facet joints, and sacroiliac joints are the main causes of CLBP [2]. Unlike lumbar radiculopathy, lumbar facet pain is rarely felt above the knee [3, 4]. General clinical characteristics of lumbar facet pain are that it eases with light flexion in the low back and the pain gets more severe with weight on the facet and extension in the low back, paraspinal tenderness, pain exacerbated by extension/rotation (facet loading), and that there is no increase in pain with flexed leg lifting or coughing [5, 6]. Lumbar facet joint disease diagnosis is a sum of the combinations consisting of history, physical activity, and diagnostic imaging frequently including computed tomography (CT) and magnetic resonance imaging (MRI) scans [7, 8]. The only way to confirm facet syndrome is pain relief by diagnostic block instead of local anaesthetic of the medial branch of the dorsal ramus of the affected lumbar facet joint [8, 9]. Prevalence of lumbar facet joint pain in CLBP is 15–45% [10–12]. Repetitive chemical and mechanical stress on lumbar facet joints may cause osteoarthritis [13, 14]. It can cause inflammation and narrowing of the capsule, resulting in axial CLBP [6]. Several treatment procedures are used in the management of lumbar facet joint-related CLBP. Intra-articular injections with corticosteroids or medial branch block are traditionally used prevalently in the management of CLBP due to lumbar facet joints [15–20]. However, evidence levels of these procedures are at a low or a medium level [21]. Moreover, corticosteroids can cause severe side effects [22, 23]. Radiofrequency neurolysis of the lumbar medial branch can be used as an alternative in the management of lumbar facet joint pain [16, 24, 25]. There are two radiofrequency applications for neurolysis: pulsed radiofrequency (PRF) and conventional radiofrequency (CRF) [26–28].

In this study, we aimed at evaluating the effect of PRF and CRF applications on pain and quality of life of patients with lumbar facet joint pain.

## 2. Materials and Methods

This prospective and double-blind study was conducted on 118 patients with lumbar facet joint pain following the approval of the local ethics committee. Written informed consent forms were taken prior to any procedures on the patients. Before any procedures on the patients, information was given regarding the operation to be performed, Visual Analogue Scale (VAS) evaluation, Odom criteria, and possible complications. In Table 3 of a conducted study [29], when the VAS scores on the morning of the 1^st^ day following the PRF operation on lumbar facet joints were evaluated, it was observed that the mean ± SD of VAS values in the study was 4.43 ± 2.9 for Group 1 and 2.38 ± 2.4 for Group 2. In the power analysis software, the mean difference of the 2 groups was calculated as 2.05 when Type 1 error was 0.05 and Type 2 error was 0.20, and sampling size for 80% power was calculated as minimum *n*=28 when SD was 2.9 for Group 1 and 2.4 for Group 2. We arranged it to be *n*=75 for Group 1 and *n*=43 for Group 2. In order to be double blind, the patients who had medial branch block due to lumbar facet pain did not know whether PRF or CRF would be applied. Whether the patients were applied PRF or CRF, RF needles stayed at the same time and RF generator was not shut down. Before the application of CRF, the patients were given 0.5 mL bupivacaine via a RF needle for local anaesthesia, and the person who performed the radiofrequency operation and the person who did the evaluation were different, and thus, the person who did the evaluation did not know which procedure was applied either. Ablation applied facet joint levels of patients of included to the study were recorded.

### 2.1. Exclusion Criteria

The exclusion criteria were as follows: uncontrolled cardiovascular, hematologic, hepatic, renal, and neurologic conditions; major depression; coagulopathy or use of anticoagulants; patients with other important chronic pain, complex regional pain syndrome, fibromyalgia, rheumatoid arthritis, and chronic fatigue syndrome; alcoholics; and substance addicts.

### 2.2. Inclusion Criteria

Housewives, retired people, and teachers were included to the study. One of the groups had 2 positive blocks in medial branches with two different local anaesthetics. One of the positive blocks were determined to be 0.5 mL 2% lidocaine, and the other was determined to be 0.5 mL 5% bupivacaine with two days apart. If both blocks were positive, this indicated that the medial branch block was at the correct level and side. Positive diagnostic block meant that the pain was relieved for 2 hours with lidocaine and for 4 hours with bupivacaine (VAS < 5 pain relief during normal activities was used as the criterion for a positive response).

There were two groups: the group which had PRF, Group 1 (*n*=75), and the group which had CRF, Group 2 (*n*=43).

## 3. Procedure

The patients included in the study were diagnosed by an algology specialist in the algology clinic of our hospital, and their operations were performed in the operating room of our hospital.

The patient was placed on the operating table in the prone position, and the lumbar region was sterilized with 4% chlorhexidine and covered with sterile covers. The target medial branch was confirmed with the AP, oblique, and lateral images using fluoroscopy, and following the anaesthetizing of the target skin with subcutaneous 2% lidocaine, a 22 G RF lesion needle with 10 mm length and 5 mm active end (radiofrequency cannula, NeuroTherm, Wilmington, ABD) was moved to the medial part of the transverse process and above. The target of the radiofrequency was the juncture of the superior articular process and transverse process for L_1–4_ levels. The needle was directly directed at the dorsal ramus towards the junction of the superior articular process and the top border of the sacral crest for the L_5_ level. Confirmation of the position of the needle and demonstration that the needle did not move towards the front border of the superior articular process towards the neural foramen were ensured with the AP, oblique, and lateral images acquired using fluoroscopy. It was confirmed with the oblique image that the needle was parallel to the medial branch nerve. Following the confirmation of the appropriate position of the needle, the RF probe (reusable radiofrequency thermocouple electrode, NeuroTherm, Wilmington, USA) was inserted inside the needle. The target nerve was stimulated with sensorial stimulation via the RF lesion generator (NeuroTherm, NT 1100, Wilmington, USA). The electrode was set up to stimulate the target neuron with 1 ms and 5 Hz. The patient was asked whether he/she experienced pain similar to their normally occurring pain or pressure. If the sensorial stimulation was 0.6 V and above, the needle was repositioned and when the stimulus was under 0.6 V, the needle position was not changed. For each lesion, correct placement was confirmed using electrostimulation at 50 Hz, with concordant sensation achieved at under 0.6 V. Before lesioning, multifidus stimulation and the absence of leg contractions was verified with electrostimulation at 5 Hz.

Following the confirmation of the optimal position of the needle, the place where the needle end was located was infiltrated with 2 mL bupivacaine. The RF probe (reusable radiofrequency thermocouple electrode, NeuroTherm, Wilmington, USA) was placed inside the needle. The RF lesion generator (NeuroTherm, NT 1100, Wilmington, USA) was set up as PRF, 2 Hz frequency at 42°C temperature in a way that the pulse waves have 20 ms width and was applied for 3 minutes. The same procedure as Group 1 was applied for the placement of the needle in Group 2, and RF neurolysis was performed by applying 80°C CRF for 90 seconds. At the end of the RF procedure, after the probe was removed in both groups, 2 mg methylprednisolone was given through the RF needle for each level. This procedure was used for each segment in both groups. The algologist collecting the data and the algologist performing the operation were different for the study to be blind. The patients were not informed about which RF procedure was applied on them.

After the procedure, the patients were monitored in the post-op unit for 2 hours and were discharged after ensuring that there were no restraints. After the patients were informed regarding the VAS evaluation (the patients were given a 10 cm paper scale with numbers between 0 and 10 with 1 cm gap between and were informed that VAS = 0 was no pain and the most severe pain they could imagine was VAS = 10, Afterwards, the patients were asked to mark which gap their pain related to with a pen), they were asked to come back for follow-up when their VAS scores were 5 and above. In addition, they were asked to come for follow-up regardless of their VAS values at the 1^st^, 3^rd^, and 6^th^ month and 1^st^ and 2^nd^ year. VAS values were asked and recorded at each follow-up, and the process was repeated by applying the same procedure if VAS was 5 and above. At the end of the second year, the patients were informed about the Odom criteria and their Odom criteria were recorded.

### 3.1. Odom Criteria

1. Perfect (all symptoms are lost and can carry out daily activities without limitation)
2. Good (there are some complaints, and there is no clear limitation on the daily activity)
3. Medium (there is subjective healing, and physical activity is subjectively better)
4. Bad (there is no healing or worse)

## 4. Statistical Analysis

All statistical data were analysed with SPSS 15.0 for Windows package software. The normal distribution of the data was tested with the Shapiro–Wilk test. The data with normal distribution were compared with the independent samples *T*-test. One-way variance analysis was used in the comparison of the VAS values in the groups within themselves, and the Tukey test, a post hoc test, was used in order to find the group which caused the difference, and the data were provided as mean ± SD. The chi-square test was used in the comparison of categorical data, and the data were given as *n*%. *p* > 0.05 was accepted as statistically significant in the comparison of all data.

## 5. Results

The comparison of the patients according to age, height, weight, duration of complaint, and gender is presented in Table 1. There was no statistically significant difference when the groups were compared in terms of age, height, weight, duration of complaint time, and gender. Comparison of VAS in groups is given in Table 2. It was observed that there was no statistically significant difference between Group 1 and Group 2 when the preoperation VAS scores of the groups were compared. There was a statistically significant difference between the two groups when Group 1 and Group 2 were compared according to their 1^st^ month VAS values, and the VAS values of Group 2 were lower than Group 1 at a statistically significant level. When Group 1 and Group 2 were compared in terms of their 3^rd^ month VAS values, there was a statistically significant difference between the two groups, and the VAS values of Group 2 were lower than Group 1 at a statistically significant level. There was a statistically significant difference between the two groups when Group 1 and Group 2 were compared in terms of 6^th^ month VAS scores, and the VAS values of Group 2 were lower than Group 1 at a statistically significant level. There was a statistically significant difference between the two groups when Group 1 and Group 2 were compared in terms of 1^st^ year VAS scores and the VAS values of Group 1 were lower than Group 2 at a statistically significant level. There was a statistically significant difference between the two groups when Group 1 and Group 2 were compared in terms of 2^nd^ year VAS scores, and the VAS values of Group 2 were lower than Group 1 at a statistically significant level.

The comparison of preoperative and postoperative VAS values of Group 1 is presented in Table 3. There was a statistically significant difference between the preoperation VAS values and postoperation 1^st^, 3^rd^, and 6^th^ month and 1^st^ and 2^nd^ year VAS values when the preoperation and postoperation 1^st^, 3^rd^, and 6^th^ month and 1^st^ and 2^nd^ year VAS values of Group 1 were compared.

The statistical difference between VAS values versus months for Group 1 is given in Table 4. There was a statistically significant difference between the postoperation 1^st^ month VAS values and 6^th^ month and 2^nd^ year VAS values for Group 1, and the 1^st^ month VAS values were lower. There was a statistically significant difference between the postoperation 3^rd^ month VAS values and 6^th^ month and 2^nd^ year VAS values for Group 1, and the 3^rd^ month VAS values were lower. There was a statistically significant difference between the postoperation 6^th^ month VAS values and 1^st^ year VAS values for Group 1, and the 1^st^ year VAS values were lower. There was a statistically significant difference between the postoperation 1^st^ year VAS values and 2^nd^ year VAS values for Group 1, and the 1^st^ year VAS values were lower.

The comparison of preoperative and postoperative VAS values of Group 2 is presented in Table 3. There was a statistically significant difference between the preoperation VAS values and postoperation 1^st^, 3^rd^, and 6^th^ month and 1^st^ and 2^nd^ year VAS values when the preoperation and postoperation 1^st^, 3^rd^, and 6^th^ month and 1^st^ and 2^nd^ year VAS values of Group 2 were compared. The statistical difference between VAS values versus months for Group 2 is given at Table 5. There was a statistically significant difference between the postoperation 1^st^ month VAS values and 1^st^ and 2^nd^ year VAS values of Group 2, and the 1^st^ month VAS values were lower. The postoperation 3^rd^ month VAS values and 1^st^ and 2^nd^ year VAS values of Group 2 were compared, and the 3^rd^ month VAS values were lower compared to the 2^nd^ year VAS values.

The comparison of Odom criteria of groups is presented in Table 6. There was a statistically significant difference between the groups when the Odom criteria of the groups were compared. There was a statistically significant difference when Group 1 and Group 2 were compared in terms of Odom 1 criteria, and the number of Odom 1 criteria was higher in Group 2. There was a statistically significant difference when Group 1 and Group 2 were compared in terms of Odom 2 criteria, and the number of Odom 2 criteria was higher in Group 2. There was a statistically significant difference when Group 1 and Group 2 were compared in terms of Odom 3 criteria, and the number of Odom 3 criteria was higher in Group 1. There was a statistically significant difference when Group 1 and Group 2 were compared in terms of Odom 4 criteria, and the number of Odom 4 criteria was higher in Group 1.

The distribution of patients' professions to the groups is given in Table 7. When comparing the Group 1 and Group 2 patients by their professions, any significant statistical difference between two groups has not been observed.

The distribution of RF levels to the groups is presented in Table 8.

The same operation was repeated for 13 patients at the 1^st^ month in Group 1. The operation was repeated for 13 patients at the 3^rd^ month in Group 1. The operation was repeated for 67 patients at the 6^th^ month in Group 1. The operation was repeated on 9 patients at the 1^st^ year in Group 1. The operation was repeated for 56 patients at the 2^nd^ year in Group 1.

The operation was repeated for 3 patients at the 1^st^ month in Group 2. The operation was repeated for 1 patient at the 3^rd^ month in Group 2. The operation was repeated on 1 patient at the 6^th^ month in Group 2. The operation was repeated for 5 patients at the 1^st^ year in Group 2. The operation was repeated for 4 patients at the 2^nd^ year in Group 2.

Bilateral medial branch PRF neurolysis was performed on 38 patients in Group 1. Bilateral medial branch CRF neurolysis was performed on 17 patients in Group 2. While medial branch PRF neurolysis was performed for a total of 322 levels in Group 1, CRF neurolysis was performed for 167 levels in Group 2. Medial branch PRF neurolysis was performed from the right side in 20 patients and from the left side in 16 patients in Group 1. CRF neurolysis was performed from the right side in 17 patients and from the left side in 14 patients in Group 2.

There was no operation on 7 patients in Group 1 as there was no sensorial response from the related facet level to sensorial stimulus, and these patients were excluded from the study. There was no operation on 8 patients in Group 2 as there was no sensorial response from the related facet level to sensorial stimulus, and these patients were excluded from the study.

In Group 1, 10 patients were excluded from the study as the pain in the test block was not relieved with local anaesthesia. In Group 2, 14 patients were excluded from the study as the pain in the test block was not relieved with anaesthesia.

2 patients in Group 1 developed neuropathic pain after 3 repetitions. Neuropathic pain therapy was initiated for these patients, and the patients were excluded from the study.

1 patient in Group 2 developed neuropathic pain after 2 repetitions. Neuropathic pain therapy was initiated for this patient, and the patient was excluded from the study. No other complications and side effects developed in the patients.

## 6. Discussion

Radiofrequency therapy has been used for 30 years in pain syndromes caused by cervicogenic headache, medulla spinalis injuries, intercostal neuralgia, back pain due to facet joint dysfunction, discogenic pain, and sacroiliac joint pain [30, 31].

There are two radiofrequency applications for neurolysis: PRF and CRF, which is the standard one such as the thermocoagulation of the dorsal ramus medial branch. The 2^nd^ is the PRF used in chronic neuropathy or radiculopathies in the treatment of dorsal root ganglion, pain trigger points, painful joints, and peripheral neuropathies [32].

Usually, CRF stimulation leads to heat lesion in the nerve material above 45°C resulting in the nonselective damage of the myelinated and unmyelinated nerve fibres [33]. PRF has a different effect mechanism consisting of the combination of other neurobiological effects [34]. Conventional radiofrequency treatment includes continuous stimulation and results in the ablation of tissues and nerves. Ablation is formed by the heat dissipating from the needle catheter [35]. Each lumbar facet joint receives innervation from the medial branches of the dorsal rami at their levels or the level above [35, 36]. At the L_1–4_ levels, the medial branch bears a constant relationship to the bone where it runs across the root of the superior articular process, and an appropriate target point, then, is the dorsal surface of the root of the transverse process immediately below the most medial end of its superior edge. The nerve is certainly within 5 mm of the point [37]. At the L_5_ level, the medial branch is not suitable for percutaneously radiofrequency neurotomy. At the L_5_ level, the dorsal ramus is the target. The target point for this nerve is where it runs along the groove between the ala of the sacrum and the root of the superior articular process [37]. As it was stated in the study of Bogduk and Long [37] regarding the anatomy of facet joint nerves, in company with fluoroscopy, we directed our needle differently for L_1–4_ and L_5_. We applied joint neurotomy to medial and lateral branches by targeting the dorsal ramus for L_5_. Currently, the “gold standard” for treating facetogenic pain is radiofrequency treatment (1 B+) [38]. The strongest indicator for lumbar facet pain is pain reduction after anaesthetic blocks of the rami mediales (medial branches) of the rami dorsales that innervate the facet joints. Because false-positive and, possibly, false-negative results may occur, the results must be interpreted carefully [38]. In our study, some patients did not respond enough to the diagnostic block, and these patients were excluded from the study. We only included patients who positively responded to the diagnostic block. Spine innervation society developed an algorithm which requires the confirmation of the healing of facet joint pain with the application of two diagnostic medial branch blocks using different local anaesthetics in each block [36]. If medial branch block is performed on the correct lumbar facet joint of the patient, the pain will be completely relieved after local anaesthetics [36]. In a cohort study by Dreyfuss et al. [3] in which a successful medial branch block and correct needle placement was performed, it was shown that medial branch nerve radiofrequency neurolysis of lumbar facet joints showed at least 80% healing in 60% of the patients at the 12^th^ month or 60% healing in 80% of the patients at the 12^th^ month.

In our study, effectiveness of RF in patients both in Group 1 and Group 2 were consistent with this study. However, frequent repetition of the PRF operation was required in Group 1 which underwent PRF (the operation was repeated for 13 patients in Group 1 at the 1^st^ month, for 13 patients in Group 1 at the 3^rd^ month, for 67 patients in Group 1 at the 6^th^ month, for 9 patients in Group 1 at the 1^st^ year, and for 56 patients in Group 1 at the 2^nd^ year), and after a certain period, the operation had to be repeated as the VAS values went above 4 in some of the patients. On the contrary, the repetition of the CRF operation was required for very few patients (the operation was repeated for 3 patients at the 1^st^ month in Group 2, for 1 patient in Group 2 at the 3^rd^ month, for 1 patient in Group 2 at the 6^th^ month, for 5 patients in Group 2 at the 1^st^ year, and for 4 patients in Group 2 at the 2^nd^ year). This suggests that PRF does not form lesions fully, and it leads to neurolysis in nerves but CRF results in neurolysis in the nerve, and this leads to a more permanent pain relief. The VAS values of 78% of the patients were below 5 at the 1^st^ year in Group 1, and the VAS values of 88.38% of the patients were below 5 at the 1^st^ year in Group 2. However, in Group 1, these rates were reached by the repetition of the operation.

Standard procedure in the management of lumbar facet joint pain is to create a lesion with the consistent heat production of the CRF ablation at 80°C for 60–90 seconds [26, 27]. This indicates the formation of the maximal thermal coagulation of the lumbar medial branch of dorsal ramus [4]. One of the potential side effects of CRF is painful skin dysesthesia and increase of pain during neurite or neurogenic inflammation [8]. This pain can cause discomfort and may require prescription of nonopioid or opioid medication for postprocedural pain management. Pain treatment can be used prevalently in postprocedural pain treatments. In our study, 1 patient in Group 2 developed neuropathic pain, and the treatment was initiated. Nonsteroid anti-inflammatory medication was initiated for all patients after the operation, and the patients were asked to use the medication for 5 days. No patient developed.

Further studies are being conducted for the availability of alternative options with fewer side effects and due to risk factors. Unlike CRF, PRF uses a short stimulation phase following a long resting phase. PRF subjects the target nerve and tissue to an electrical field, and this very rarely causes damage on these structures [28]. However, the mechanism of PRF is not fully understood. The electrical field produced by PRF can change pain signals and has selective effect on small unmyelinated fibres (C fibres) [37, 38]. Currently, PRF is used in different types of pain such as neuralgia, joint pain, and myofascial pain [39–41]. It was reported that PRF stimulation after the lumbar medial branch had a positive effect on the management of CLBP [8]. Additionally, PRF stimulation after the placement of the needle electrode in the joint cavity can effectively decrease resistant joint pain [42–45]. However, there is very little known about the effects of intra-articular PRF stimulation in the management of lumbar facet joint pain. Neuropathic pain developed in 2 patients in Group 1, and treatment was initiated, but postoperation anti-inflammatory medication treatment was not initiated for any of the patients due to the operation. In our study, PRF was performed on the medial branch of the patients with lumbar facet joint pain in Group 1, and it was observed to be effective though for a short time. However, its effectivity was only ensured by repetition when VAS values dropped below 5.

There was no significant statistical difference between the distributions of patients to the groups based on their professions. However, while six patients in Group 1 affirmed the Odom 1 criteria, 16 patients in Group 2 affirmed the Odom 1 criteria. The Odom 2 criterion was affirmed by 2 patients in Group 1 and 22 patients in Group 2. The satisfaction level of the patients at the 2^nd^ year was evaluated with the Odom criteria in our study, and it was observed that the patients in Group 2 were more satisfied compared to Group 1.

In conclusion, both PRF and CRF can be used for medial branch block in lumbar facet joint pain. PRF is effective for a short period in the medial branch block performed due to lumbar facet joint pain, but its effectivity is weaker compared to CRF. Thus, VAS values increase sooner, and more frequent RF is required. The effectivity of CRF on the medial branch block due to lumbar facet joint pain is strong and long term. There is no need for frequent RF. However, the use of postoperation anti-inflammatory medications is required due to pain associated with the operation. In the long term (at the end of the 2^nd^ year), patients who had CRF applied were more satisfied at a statistically significant level. With CRF application, the levels of quality of life and daily activities of patients were observed as the same before the lumbar facet joint pain.

## Figures and Tables

**Table 1:** Demographic data of groups (mean ± SD and *n* (%)).

	Age (years)	Height (cm)	Weight (kg)	Complaint time (months)	Gender F/M, *n* (%)
Group 1 (*n*=75)	53.90 ± 16.23	171 ± 72.9	79.92 ± 10.45	12.82 ± 4.63	43/32 (57.33%/40.67%)
Group 2 (*n*=43)	53.39 ± 16.14	168 ± 71.58	78.37 ± 12.16	14.74 ± 6.56	29/14 (67.44%/32.56%)
*p*	0.869	0.91	0.486	0.096	0.279

**Table 2:** Comparison of VAS values of the groups according to months (mean ± SD).

	Preoperation VAS values	1^st^ month VAS values	3^rd^ month VAS values	6^th^ month VAS values	1^st^ year VAS values	2^nd^ year VAS values
Group 1 (*n*=75)	8.10 ± 0.95	3.79 ± 1.05	4.09 ± 1.11	5.66 ± 1.14	3.69 ± 0.86	5.20 ± 1.06
Group 2 (*n*=43)	8.44 ± 0.93	3.27 ± 1.24	3.39 ± 1.04	3.79 ± 0.67	4.04 ± 0.81	4.09 ± 0.78
*p*	0.066	^*∗*^0.027	^*∗*^0.001	^*∗*^<0.001	^*∗*^<0.001	^*∗*^<0.001

^*∗*^Statistically significant.

**Table 3:** Comparison of preoperation VAS values and postoperation at 1^st^, 3^rd^, and 6^th^ month and 1^st^ and 2^nd^ year VAS values of Groups 1 and 2.

	Preoperation VAS values	1^st^ month VAS values	3^rd^ month VAS values	6^th^ month VAS values	1^st^ year VAS values	2^nd^ year VAS values	*p*
Group 1 (*n*=75)	8.10 ± 0.95	3.79 ± 1.05	4.09 ± 1.11	5.66 ± 1.14	3.69 ± 0.86	5.20 ± 1.06	^*∗*^<0.001
Group 2 (*n*=43)	8.44 ± 0.93	3.28 ± 1.24	3.42 ± 1.05	3.77 ± 0.68	4.04 ± 0.81	4.09 ± 0.78	^*∗*^<0.001

^*∗*^Statistically significant.

**Table 4:** Results of the Tukey test from post hoc tests showing the comparison of preoperation and postoperation at 1^st^, 3^rd^, and 6^th^ month and 1^st^ and 2^nd^ year VAS values of Group 1 among themselves (*p* values).

	1^st^ month VAS values	3^rd^ month VAS values	6^th^ month VAS values	1^st^ year VAS values	2^nd^ year VAS values
Preoperation VAS values	^*∗*^<0.001	^*∗*^<0.001	^*∗*^<0.001	^*∗*^<0.001	^*∗*^<0.001
1^st^ month VAS values		0.461	^*∗*^<0.001	0.994	^*∗*^<0.001
3^rd^ month VAS values			^*∗*^<0.001	0.173	^*∗*^<0.001
6^th^ month VAS values				^*∗*^<0.001	0.067
1^st^ year VAS values					^*∗*^<0.001

^*∗*^Statistically significant.

**Table 5:** Results of the Tukey test from post hoc tests showing the comparison of preoperation and postoperation at 1^st^, 3^rd^, and 6^th^ month and 1^st^ and 2^nd^ year VAS values of Group 2 among themselves (*p* values).

	1st month VAS values	3rd month VAS values	6th month VAS values	1st year VAS values	2nd year VAS values
Preoperation VAS values	^*∗*^<0.001	^*∗*^<0.001	^*∗*^<0.001	^*∗*^<0.001	^*∗*^<0.001
1^st^ month VAS values		0.983	0.154	^*∗*^0.002	^*∗*^0.001
3^rd^ month VAS values			0.515	^*∗*^0.025	^*∗*^0.012
6^th^ month VAS values				0.738	0.591
1^st^ year VAS values					1

^*∗*^Statistically significant.

**Table 6:** Comparison of Odom criteria of the groups (*n* (%).

		Odom 1	Odom 2	Odom 3	Odom 4
Group 1 (*n*=75)	Negative	69 (58.5%)	73 (61.9%)	48 (40.7%)	35 (29.7%)
	Positive	6 (5.1%)	2 (1.7%)	27 (22.9%)	40 (33.9%)
Group 2 (*n*=43)	Negative	27 (22.9%)	21 (17.8%)	41 (34.7%)	41 (34.7%)
	Positive	16 (13.6%)	22 (18.6%)	2 (1.7%)	2 (1.7%)
*p*		^*∗*^<0.001	^*∗*^<0.001	^*∗*^<0.001	^*∗*^<0.001

^*∗*^Statistically significant.

**Table 7:** Distribution of patients to the groups based on their professions (*n* (%)).

Groups	Housewives	Retired people	Teachers	Total	*p*
Group 1	43 (36.4%)	22 (18.6%)	10 (8.5%)	75 (63.6%)	0.284
Group 2	29 (24.6%)	7 (5.9%)	7 (5.9%)	43 (36.4%)	
Total	72 (61%)	29 (24.6%)	17 (14.4%)	118 (100%)	

**Table 8:** Distribution of RF levels to the groups (*n*).

Levels of ablation	Group 1		Group 2	
	Procedure (*n*)	Levels (*n*)	Procedur (*n*)	Levels (*n*)
Right L_2–5_	5	20	2	8
Left L_2–5_	3	12	4	16
Bilateral L_2–5_	8	64	3	24
Right L_3-4_	9	18	4	8
Left L_3-4_	8	16	3	6
Bilateral L_3-4_	12	48	5	20
Right L_3–5_	7	21	6	18
Left L_3–5_	5	15	7	21
Bilateral L_3–5_	18	108	9	54
Total	75	322	43	167

## Data Availability

The data used to support the findings of this study are available from the corresponding author upon request.
